# Minimal residual disease after transplantation or lenalidomide-based consolidation in myeloma patients: a prospective analysis

**DOI:** 10.18632/oncotarget.12641

**Published:** 2016-10-13

**Authors:** Stefania Oliva, Manuela Gambella, Milena Gilestro, Vittorio Emanuele Muccio, Francesca Gay, Daniela Drandi, Simone Ferrero, Roberto Passera, Chiara Pautasso, Annalisa Bernardini, Mariella Genuardi, Francesca Patriarca, Elona Saraci, Maria Teresa Petrucci, Norbert Pescosta, Anna Marina Liberati, Tommaso Caravita, Concetta Conticello, Alberto Rocci, Pellegrino Musto, Mario Boccadoro, Antonio Palumbo, Paola Omedè

**Affiliations:** ^1^ Myeloma Unit, Division of Hematology, University of Torino, Azienda Ospeadliero-Universitaria Città della Salute e della Scienza di Torino, Torino, Italy; ^2^ Division of Hematology, Department of Molecular Biotechnologies and Health Sciences, University of Torino, Torino, Italy; ^3^ Division of Nuclear Medicine, University of Torino, Azienda Ospedaliero-Universitaria Città della Salute e della Scienza di Torino, Torino, Italy; ^4^ Azienda Ospedaliera-Universitaria di Udine, DISM Università di Udine, Udine, Italy; ^5^ Division of Hematology, Department of Cellular Biotechnologies and Hematology, Sapienza University of Rome, Rome, Italy; ^6^ Ematologia e Centro TMO, Ospedale Centrale Bolzano, Bozen, Italy; ^7^ AO S.Maria di Terni, SC Oncoematologia, Terni, Italy; ^8^ UOC Ematologia S.Eugenio ASL RM2 Roma, Rome, Italy; ^9^ Divisione di Ematologia, Azienda Policlinico-OVE, Università di Catania, Catania, Italy; ^10^ Department of Haematology, Manchester Royal Infirmary, Central Manchester University Hospitals NHS Foundation Trust, Manchester, UK; ^11^ Scientific Direction, IRCCS-CROB, Referral Cancer Center of Basilicata, Rionero in Vulture (Pz), Italy

**Keywords:** myeloma, MRD, ASO-RQ-PCR, novel agents, flow cytometry

## Abstract

We analyzed 50 patients who achieved at least a very good partial response in the RV-MM-EMN-441 study. Patients received consolidation with autologous stem-cell transplantation (ASCT) or cyclophosphamide-lenalidomide-dexamethasone (CRD), followed by Lenalidomide-based maintenance. We assessed minimal residual disease (MRD) by multi-parameter flow cytometry (MFC) and allelic-specific oligonucleotide real-time quantitative polymerase chain reaction (ASO-RQ-PCR) after consolidation, after 3 and 6 courses of maintenance, and thereafter every 6 months until progression. By MFC analysis, 19/50 patients achieved complete response (CR) after consolidation, and 7 additional patients during maintenance. A molecular marker was identified in 25/50 patients, 4/25 achieved molecular-CR after consolidation, and 3 additional patients during maintenance. A lower MRD value by MFC was found in ASCT patients compared with CRD patients (p=0.0134). Tumor burden reduction was different in patients with high-risk vs standard-risk cytogenetics (3.4 vs 5.2, ln-MFC; 3 vs 6 ln-PCR, respectively) and in patients who relapsed vs those who did not (4 vs 5, ln-MFC; 4.4 vs 7.8 ln-PCR). MRD progression anticipated clinical relapse by a median of 9 months while biochemical relapse by a median of 4 months. MRD allows the identification of a low-risk group, independently of response, and a better characterization of the activity of treatments.

## INTRODUCTION

Multiple Myeloma (MM) is an incurable hematological disease. Nevertheless, the availability of novel agents, incorporated into pre-transplant and post-transplant consolidation and/or maintenance strategies, strongly improved outcome and response rates [1, 2]. Several studies have demonstrated a direct correlation between depth of response to front-line therapy and prolonged survival [3–6].

Moreover, newer outcome measures and the definition of specific subcategories of complete response (CR) with different degrees of stringency allow a more precise definition of response, a better comparison of efficacy between treatments, and a more accurate detection and monitoring of relapse. In this regard, the International Myeloma Working Group (IMWG) introduced stringent CR (sCR) criteria by adding the normalization of serum free-light chain and the absence of clonal plasma cells to the classical definition of CR [7]. Minimal residual disease (MRD) assessment by multiparameter flow cytometry (MFC) and allelic-specific oligonucleotide real-time quantitative polymerase chain reaction (ASO-RQ-PCR) on bone marrow (BM) is a sensitive strategy to accurately measure response [8–13] and has been recently included in the IMWG criteria (immunophenotypic and molecular CR, respectively) [14]. The achievement of MRD negativity is highly predictive of outcome in MM both in transplant-eligible and -ineligible patients [14].

The aim of this study is to evaluate the role of MRD monitoring by MFC and ASO-RQ-PCR and its impact on outcome of MM patients receiving novel agents with or without autologous stem cell transplantation (ASCT), and to evaluate the role of MRD during maintenance therapy.

## RESULTS

### Patients characteristics and response evaluation

The MRD sub-study started after approximately 2 years from the start of the clinical study, therefore only 54 out of 134 patients enrolled in the main study who achieved at least a very good partial response (VGPR) after consolidation were included [15]. Baseline clinical characteristics of patients in the sub-study were comparable to those with at least a VGPR in the main study (data not shown). Four patients were excluded because they had polyclonal immunophenotype. After consolidation, 34 patients (68%) achieved a VGPR (16 received Cyclophosphamide-Lenalidomide-Dexamethasone [CRD] vs 18 ASCT); 16 (32%) a CR (9 received CRD vs 7 ASCT) (Appendix Figure 1). Median age was 57 years (IQR 54 to 61 years); 8 patients (16%) had International Staging System (ISS) stage III and 11 (22%) had high-risk cytogenetic profile [at least one chromosomal abnormalities among deletion 17p, t (4;14) and t (14;16)] (Table 1). Twenty-five patients out of 50 (50%) received ASCT and 25 (50%) received CRD consolidation. All 50 patients started maintenance: 27 (54%) with Lenalidomide and Prednisone, 23 (46%) with Lenalidomide alone.

**Figure 1:**
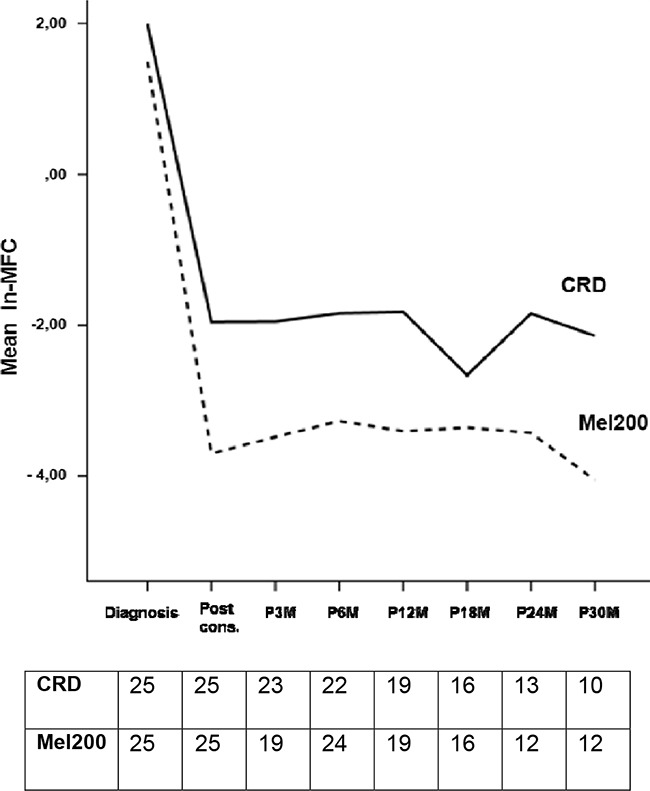
Observed marginal means of ln-MFC values according to consolidation regimen

**Table 1:** Demographic and baseline characteristics of the MRD sub-study group

	MRD sub-study (n=50)
**Age (years)**	
**Median**	57
**IQR**	54-61
**Gender N (%)**	
**Male**	23 (46%)
**ISS Stage N (%)**	
**I**	27 (54%)
**II**	15 (30%)
**III**	8 (16%)
**Creatinine (mg/dL)**	
**Median**	0,9
**IQR**	0.73-1.10
**Missing data N (%)**	2 (4%)
**LDH (U/L)**	
**Median**	321
**IQR**	237-386
**Missing data N (%)**	12 (24%)
**Hemoglobin (g/L)**	
**Median**	11,8
**IQR**	10.60-12.43
**Missing data N (%)**	1 (2%)
**Cytogenetic features N (%)**	
**Deletion 17p**	3 (6%)
**Translocation (4;14)**	5 (10%)
**Translocation (14;16)**	4 (8%)
**High-risk**	11 (22%)
**Missing data**	8 (16%)
**Random**	
**CRD**	25 (50%)
**Mel200**	25 (50%)

IQR: Interquartile range, ISS: International Staging System, LDH: lactate dehydrogenase, CRD: Cyclophosphamide, Lenalidomide, Dexamethasone, Mel200: Melphalan 200 mg/m^2^

At data cut-off, the median duration of maintenance was 27.2 months (IQR 16-34), 5 patients discontinued maintenance due to adverse events, including 1 with a second cancer.

After a median follow-up of 57 months from enrollment, 27 (54%) progressions and 9 (18%) deaths were recorded, with a 5-year progression-free survival (PFS) of 45% and a 5-year overall survival (OS) of 80%.

### MRD assessment by MFC

**Figure 2:**
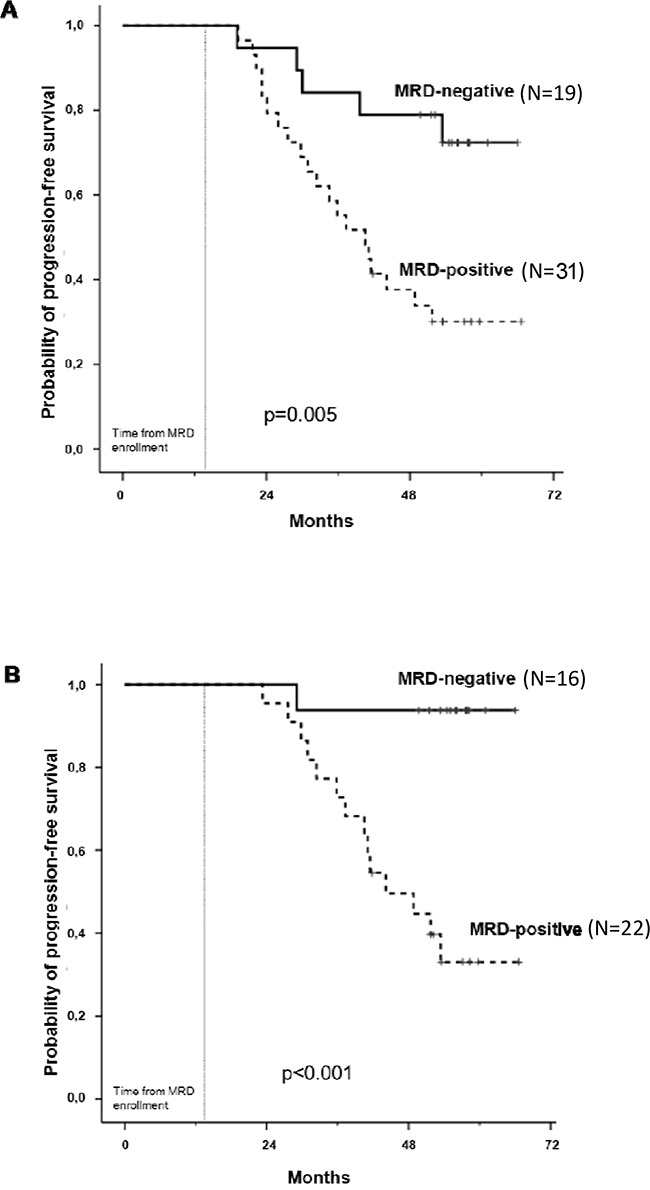
Progression-free survival analysis by MFC after consolidation A. and 1 year of maintenance B

**Figure 3:**
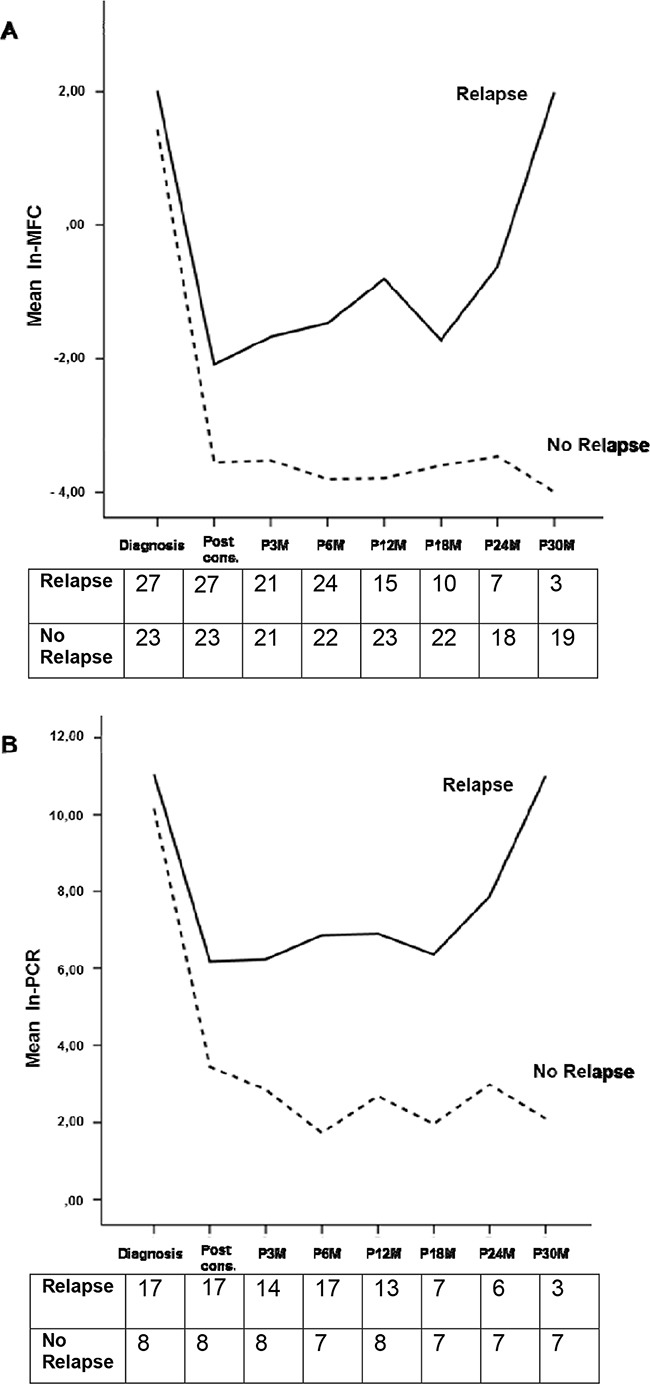
Observed marginal means of ln-MFC A. and ln-PCR B. values according to relapse or no relapse Tables report the analyzed cases at each time-point for each group (relapse and no relapse)

In the MFC analysis, 50 patients have been evaluated: 19 patients (38%) achieved MRD-negativity after consolidation, including 12 patients in the ASCT group and 7 in the CRD group (63% vs 37%, respectively). Patients receiving ASCT showed a lower value of residual cells (median 0.002%, range 0 – 1%) compared with patients receiving CRD (median 0.2%, range 0 – 2.9%, p=0.0134) and this was confirmed using the time series plots of the log- transformed repeated measures (5.1 vs 3.9 ln-MFC tumor burden reduction, respectively): a deeper MRD shrinkage (approximately 2 log units) was detected at consolidation in the ASCT group vs the CRD group. Subsequently, MRD values remained stable in both subgroups (Figure 1). After consolidation, 5-year PFS was 72% for MRD negative vs 30% for MRD positive patients (p=0.005) (Figure 2A). Importantly, patients who experienced clinical relapse showed different tumor burden kinetics after consolidation (4 ln-MFC tumour burden decrease) when compared with patients who did not relapse (5 ln-MFC tumour burden decrease): specifically, approximately 1.5 log units of higher tumor shrinkage were observed in patients with favorable outcome (Figure 3A); a progressive MRD increase in the following MRD time points was seen in patients who relapsed in contrast to a substantially stable disease in patients who did not relapse. In addition, unfavorable tumor kinetics was observed in patients with high-risk cytogenetic (3.4 ln-MFC reduction) vs standard-risk (5.2 ln-MFC reduction) (Appendix Figure 2A). In the PFS analysis, we observed a significant difference in patients with high-risk cytogenetics and MRD negativity after consolidation compared with patients with standard-risk cytogenetics and MRD negativity: median PFS was 24,1 months vs not reached (p=0.03), respectively.

During maintenance 7 additional patients (14%) achieved MRD negativity: 2 in the Lenalidomide-Prednisone group and 5 in the Lenalidomide alone group. In particular, 4 upgraded their MRD response after 3 months of starting maintenance, 2 after 6 months and 1 after 24 months. Of these, 5 received ASCT consolidation. Overall among the 26 patients who were MRD negative, 8 (31%) relapsed; whereas among the 24 patients who did not obtain MRD negativity, 19 (79%) relapsed. In all patients who achieved MRD negativity, the median PFS was not reached vs 35 months in those who did not achieve MRD negativity (p=0.0004).

**Figure 4:**
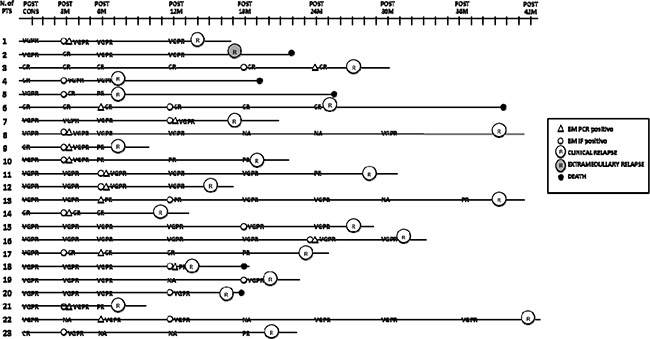
Evaluation of immunophenotypic, molecular and biochemical relapse and correlation with clinical relapse Post cons: after consolidation, post 3M: after 3 months of maintenance, post 6M: after 6 months of maintenance, post 12M: after 12 months of maintenance, post 18M: after 18 months of maintenance, post 24M: after 24 months of maintenance, post 30M: after 30 months of maintenance, post 36 M: after 36 months of maintenance, post 42 M: after 42 months of maintenance; VGPR: very good partial response, CR: complete response, R: clinical relapse, ER: extramedullary relapse, NA: not available, IF: immunophenotypic; PTS: patients.

Twenty-three out of 27 relapsing patients were accurately monitored for MRD, while 4 patients could not be evaluated due to the lack of samples. In 22/23 patients, MFC progression anticipated clinical relapse by a median of 9 months (IQR 4-17 months), while in one patient clinical extramedullary progression anticipated MFC and biochemical progression. On the other hand, in 10/23 patients, biochemical relapse or relapse from CR anticipated clinical relapse by a median of 4 months (IQR: 2-10) (Figure 4)

.

PFS analysis was performed at different time-points during maintenance and the 5-year PFS was 94% for MRD negative vs 33% for MRD positive patients at 12 months of maintenance (p<0.001) (Figure 2B).

### MRD assessment by ASO-RQ-PCR

A specific IgH molecular marker was identified in 25 patients (50%) and they were monitored for MRD: 14 (28%) had a BM infiltration rate at diagnosis lower than 5% and 11 out of 50 (22%) did not obtain a successful sequencing, due to the elevated somatic hypermutation (SHM) rate of the immunoglobulin gene loci.

ASO-RQ-PCR results showed that overall 7 patients out of 25 achieved a molecular CR: 4 (16%) after consolidation, 3 (12%) during Lenalidomide maintenance. Among the 7 patients who achieved molecular CR, 2 (28%) progressed after a median of 34.4 months (1 patient became MRD positive and relapsed and 1 patient had a clinical extra-medullary progression). Among the 18 patients who did not achieve a molecular CR, 15 (83%) progressed after a median of 35.8 months (p=0.02). In 16 out of 17 patients who experienced disease progression, molecular-progression anticipated clinical relapse by a median of 10 months (IQR 5-19) confirming MFC results.

Similarly to the MFC analysis, different tumor burden kinetics were observed among patients who relapsed vs patients who did not (4.4 vs 7.8 ln-PCR reduction, respectively) (Figure 3B), high-risk cytogenetic vs standard-risk groups (3 vs 6 ln-PCR reduction, respectively) (Appendix Figure 2B) with approximately 3 log units of higher tumor shrinkage for patients with favorable outcome. Due to the limited patient series evaluable by ASO-RQ-PCR, no PFS analysis could be performed.

## DISCUSSION

In this prospective analysis, we monitored MRD using both MFC and ASO-RQ-PCR methods in 50 newly diagnosed MM patients who achieved at least a VGPR after ASCT or CRD consolidation, followed by Lenalidomide maintenance. Although in this sub-study the VGPR rate was equal in the CRD and ASCT arms, we found a deeper MRD shrinkage with ASCT vs CRD consolidation, confirming the clinical advantage of ASCT [15]. Moreover, the prognostic role of MRD was confirmed, with a 5-year PFS after consolidation of 72% vs 30% for MRD-negative and MRD-positive patients, respectively (P=0.005). These results suggest that in CR/VGPR patients a deeper characterization of response is needed in order to detect residual tumor cells and possibly to apply an MRD-driven approach. Importantly, BM evaluation, both by MFC and ASO-RQ-PCR, allowed anticipating clinical relapse by a median of 9-10 months (thus confirming our previous reports) [16] and biochemical relapse by electrophoretic evaluation by a median of 4 months. Therefore, MRD analysis could help to promptly identify relapsing patients with unsustained responses. Moreover, Lenalidomide maintenance was able to maintain MRD shrinkage and, in some patients, increased depth of response, from MRD positive to MRD negative status. This may be due to a delayed effect of transplant, considering that 3 patients became MRD negative after 3 months and 2 after 6 months. Nevertheless, the median time to MRD negativity from transplant was 8 months, supporting the idea that also an effect of lenalidomide could influence tumor burden in this population. We could not evaluate the possible difference between Lenalidomide-Prednisone and Lenalidomide alone arms due to the limited number of cases.

The detection and prognostic role of MRD in myeloma patients showed to be of great interest in the past ten years. Several techniques and new methodologies are emerging to define response to therapy more rigorously, making the scenario very confusing [17]. Serological biomarkers, such as immunofixation or immunoglobulin free-light chains, are widely available today, and have the advantage of being non-invasive procedures [18]. Of note, they allow the diagnosis and monitoring of MM, and they improve risk stratification [19–21]. However, results from available studies are contradictory and the incorporation of serum free-light chains in the measurement of MRD is still controversial [22–25]. MFC and ASO-RQ-PCR are sensitive approaches to detect MRD in the BM of MM patients receiving novel agents and/or ASCT [26]. To the best of our knowledge, this is the first MRD study comparing transplant vs no transplant MRD negativity by MFC and ASO-RQ-PCR.

In two PETHEMA/GEM trials (GEM2000 and GEM2005<65y), risk assessment by FISH and MRD monitoring by flow cytometry had an independent prognostic value in transplant-eligible patients [8]. Conversely, Rawstron et al. confirmed that the presence of MRD was a strong predictor of outcome in patients with both favorable and adverse cytogenetic profile [27].

In our study, high-risk cytogenetic profile by FISH was associated with lower reduction of tumor burden and higher rates of MRD reappearance when compared with standard-risk cytogenetic profile. Consistently with results reported by the Spanish group [28], these data suggest that combining cytogenetics and MRD data could be a valid option to identify a subset of CR/VGPR patients with a worse outcome and who should be candidates for novel treatment strategies.

The present MRD sub-study was started 2 years after the beginning of the clinical trial, thus a low number of patients was included (50 for MFC and 25 for ASO-RQ-PCR). This could be a limitation to the sub-study, which has also hampered an extensive comparison between the two methods.

Some technical issues regarding both MRD methods also need to be considered. ASO-RQ-PCR in MM is a complex and time-consuming technique. Moreover, despite the high sensitivity (up to 10^-5^) of this method, currently 40-50% of patients lack a molecular marker due to the elevated SHM rate of the immunoglobulin gene loci, thus negatively affecting sequencing. Several studies employed a mono-targeted strategy (IGH); the complexity in detecting and sequencing Ig target in low infiltrated baseline samples (<5% of plasma cells) is largely known and the low quality of samples, deriving from multicenter trials, might hamper the applicability of this technique. MFC seems to be more easily applicable and does not require patient-specific diagnostic phenotypic profiles. The need for high quality BM sampling, the lack of standardization of different protocols, and the variability of technique sensitivity and data interpretation among different laboratories are some of the major drawbacks of MFC [17, 26]. In light of these considerations, our study showed the higher feasibility of MFC compared with ASO-RQ-PCR due to the low marker detection rate and the complex profile of the molecular technique.

In order to overcome these limitations, newer strategies have been tested such as Next Generation Sequencing (NGS) by using the LymphoSIGHT platform, which allows the amplification and sequencing of all rearranged immunoglobulin gene segments present in myeloma clone, reaching a sensitivity of 10^-6^ or better [29–31]. In a Spanish trial, MRD negative status was associated with significantly longer time to progression (median 80 vs 31 months, P<0.001) and OS (median not reached vs 81 months, P=0.02) [30].

Unlike other hematological disorders, BM infiltration pattern is not uniform in MM and hemodiluited BM aspirates may lead to false-negative results. MM presents high frequency of extramedullary relapses and sensitive imaging techniques have become relevant in assessing low levels of disease outside BM [32–35]. In our study, one patient with BM negative MRD experienced extramedullary relapse, confirming the importance of imaging techniques in patients with MRD negativity. A recent Italian retrospective analysis also confirmed the prognostic role of PET/CT when performed at baseline in combination with ISS stage. Furthermore, PET/CT showed to be a valuable technique to improve the definition of CR after treatment and to detect otherwise unidentifiable progression in the follow-up phase of the disease [36].

In conclusion, this sub-study showed that persistence of MRD is an adverse prognostic factor, even among CR or VGPR patients. In addition, MRD monitoring during and after treatment should be considered at least in the context of clinical trials as a more sensitive approach compared with routine evaluation, in order to explore MRD-guided therapeutic decisions. Indeed, MRD may become a valuable tool for clinicians to understand which patients may benefit from continuous treatment and which ones may be considered for an alternative strategy. Moreover, future analyses on larger cohorts are needed to overcome the current limitations associated with repeated bone marrow assessments. New MRD strategies using peripheral blood may be a valid alternative [37, 38], and new, more sensitive techniques such as NGS may play a crucial role.

## METHODS

The RV-MM-EMN-441 trial was a multicenter, randomized phase III trial (ClinicalTrials.gov identifier: NCT01091831) [15]. The main study was approved by the institutional review boards of each participating center, and was conducted according to the Declaration of Helsinki. All patients provided written informed consent.

### Patients and study design

**Figure 5:**
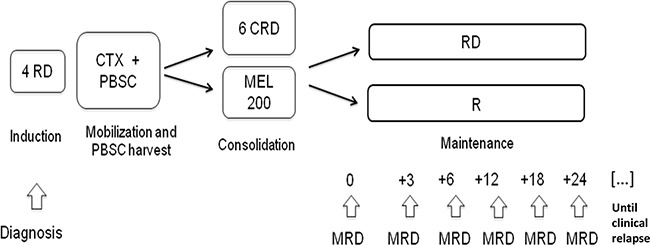
Study design and MRD time-points RD: Lenalidomide-Dexamethasone, CTX: Cyclophosphamide, PBSC: peripheral blood stem cell, CRD: Cyclophosphamide-Lenalidomide-Dexamethasone, MEL200: Melphalan 200 mg/m^2^, R: Lenalidomide, MRD: minimal residual disease.

Results from the main study have been recently reported [15], and the study protocol is summarized in Figure 5. Briefly, all patients received four cycles of Lenalidomide+low-dose Dexamethasone (RD) as induction, followed by mobilization of BM stem cells using Cyclophosphamide and Granulocyte-Colony Stimulating Factor (G-CSF), and stem cell collection. Subsequently patients received either consolidation with six cycles of CRD or high-dose Melphalan and ASCT. After the consolidation phase, patients were further randomized to maintenance with either Lenalidomide or Lenalidomide-Prednisone until relapse or intolerance. Response to treatment was assessed according to the IMWG criteria [14]. Patients who achieved at least a VGPR after consolidation were eligible for the present MRD sub-study. Biochemical relapse was defined as an increase of 25% from lowest response value in any of the following: serum M-component (absolute increase ≥0.5 g/100 ml) and/or urine M-component (absolute increase ≥200 mg per 24 hours); relapse from CR was defined as the reappearance of serum or urine M-protein by immunofixation or electrophoresis.

MRD analysis was performed on BM aspirates collected at different time-points: after consolidation, after 3 and 6 courses of maintenance, and then every 6 months until clinical relapse.

### MRD assessment by multiparameter flow cytometry

MFC analysis was performed in a centralized laboratory (Laboratory of Cytofluorimetry-University of Turin, Italy). Plasma cells phenotypic aberrations were identified at diagnosis and were used as patient-specific profile in the follow-up analyses. BM cells for MRD detection by MFC were lysed following the EuroFlow recommendations [39].

The following monoclonal antibodies (MoAbs) combinations were employed:

1) CD138FITC/CD56PE/CD20PerCp/CD117APC/CD45APC-H7/CD38PE-Cy7

2) cyKappaFITC/cyLambdaPE/CD19PerCp/CD56APC/CD45APC-H7/CD38PE-Cy7.

From the first combination, we obtained plasma cells quantification; from the second combination, we evaluated plasma cells immunophenotype and clonality. Acquisition and analyses were performed using FACSCanto II (BD Biosciences, San Josè, CA) and DiVa software: a minimum of 1-2 × 10^6^ of events for each sample were acquired.

MFC analysis had a sensitivity of 10^−4^ cells. MFC-CR was defined as <0.01% monoclonal plasma cells in the BM sample confirmed on two consecutive BM assessments. MFC relapse was defined by confirmed 25% increase of malignant plasma cells in two subsequent samples.

### MRD assessment by allelic-specific oligonucleotide real-time quantitative polymerase chain reaction

Genomic DNA from total white blood cells of BM samples was isolated using DNAzol reagent (Life Technologies-Invitrogen, Carlsbad, CA, USA), following the manufacturer's instructions. Patient-specific IgH rearrangements were amplified and directly sequenced from genomic DNA at diagnosis [40]. Sequences were analyzed by IMGT/V-QUEST tool [41, 42], and patient-specific ASO-primers and consensus probes were designed as previously described [40]. IgH-based MRD detection by ASO-RQ-PCR was performed using an AbiPrism7900HT (Life Technologies-Applied Biosystems, Carlsbad, CA, USA) [17]. MRD analysis was interpreted following the Euro-MRD guidelines [43].

Moreover, molecular-CR was defined as two consecutive negative MRD results by ASO-RQ-PCR with minimal sensitivity of 10^−5^. Molecular progression was defined as the reappearance of positivity or a confirmed 25% increase of ASO-RQ-PCR values in two repeated follow-up samples.

### Statistical methods

The primary endpoint was to investigate MRD response rates by using MFC and ASO-RQ-PCR methods, at different time-points. Secondary endpoints were the duration of MRD response and progression-free survival (PFS) stratified on MRD response. PFS was defined as the time from start of treatment to documented progression or death from myeloma; patients still alive were censored at last contact date. PFS was analyzed by the Kaplan-Meier method, comparing the two groups by the log-rank test and calculating 95% confidence intervals for the following covariates: gender (female vs. male), age at diagnosis (56+ yrs vs. ≤55 yrs), International Staging System (ISS) stage, cytogenetics (high-risk vs. standard-risk FISH).

The main patient characteristics were tested by the Fisher's exact test for categorical variables and by the Mann-Whitney test for continuous ones. Since the natural logarithms ln-MFC and ln-PCR were not normally distributed by time-points, it was impossible to test them by the mixed linear model for repeated measures. Therefore, we compared the observed marginal means of ln-MFC and ln-PCR for each time-point, by the Friedman test (non-parametric analysis of variance for repeated measures), undertaking a post-hoc analysis by the Wilcoxon signed-rank test (non-parametric t-test for repeated measures) and adjusting the p-values by Bonferroni correction as already published [11]. All reported p-values were obtained by the two-sided exact method, at the conventional 5% significance level. Data were analyzed as of December 2015 by IBM SPSS 21.0.

## SUPPLEMENTARY MATERIALS FIGURES


