# Supplementary material for: Minimal residual disease after transplantation or lenalidomide-based consolidation in myeloma patients: a prospective analysis
Source: Oncotarget. 2016 Oct 13;8(4):5924–35. doi: 10.18632/oncotarget.12641 (PMC5351601; doi:10.18632/oncotarget.12641)
Supplement: Supplementary file 1 [file oncotarget-08-5924-s001.pdf]

Minimal residual disease after transplantation or lenalidomide-based consolidation in myeloma patients: a prospective analysis

APPENDIX FIGURES

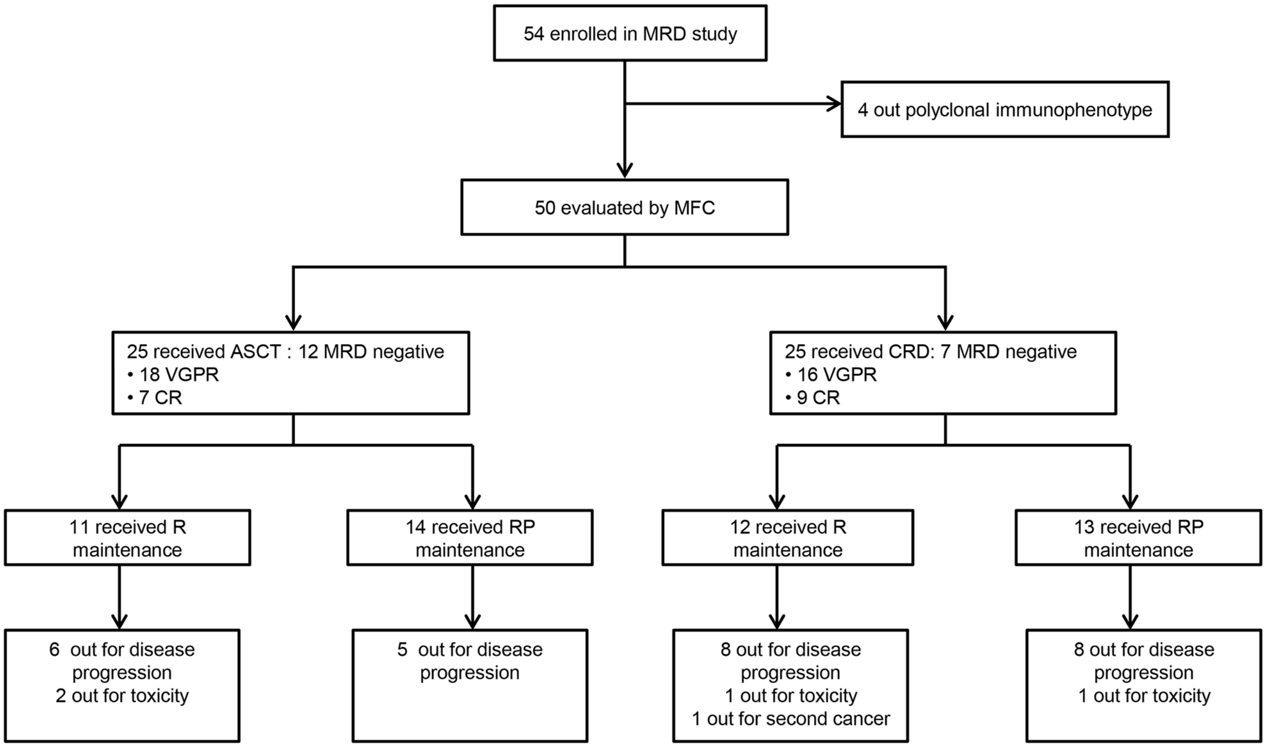

Appendix Figure 1: Flow diagram. MRD, minimal residual disease; MFC, multiparameter flow cytometry; ASCT, autologous stem cell transplantation; CRD, cyclophosphamide-lenalidomide-dexamethasone; VGPR, very good partial response; CR, complete response; R, lenalidomide; RP, lenalidomide-prednisone

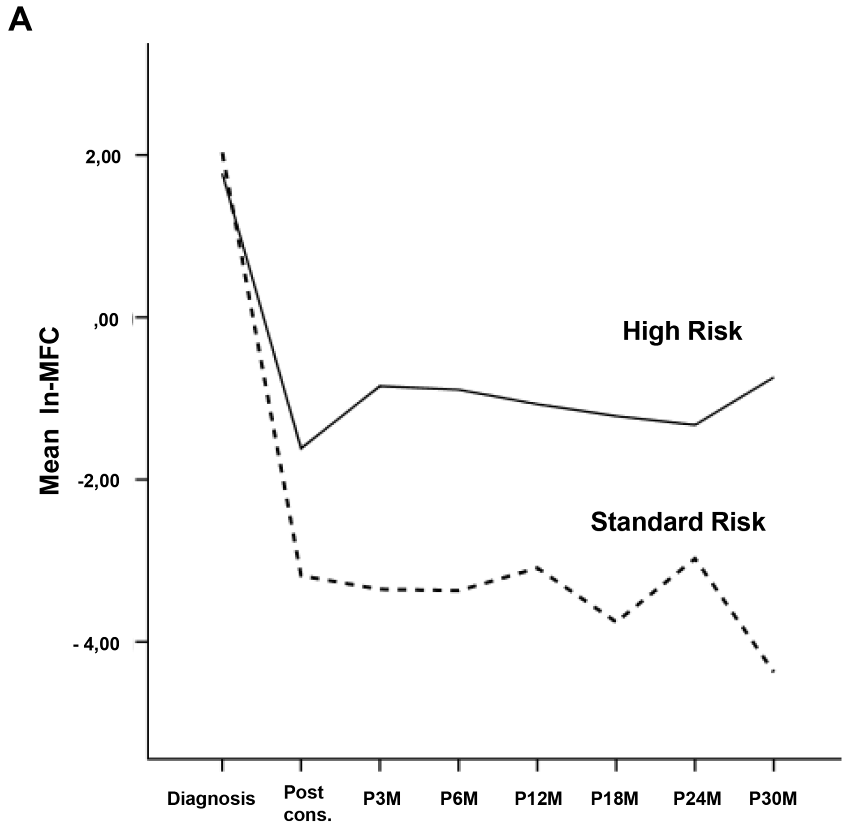

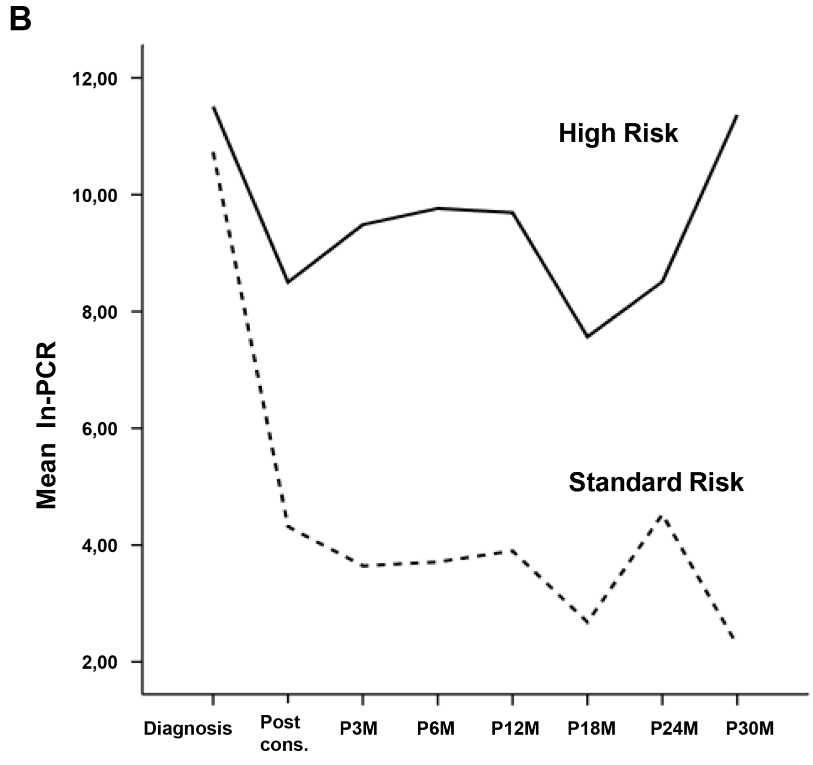

Appendix Figure 2: Observed marginal means of ln-MFC A. and ln-PCR B. values according to cytogenetic.
